# Spin Absorption Effect at Ferromagnet/Ge Schottky-Tunnel Contacts

**DOI:** 10.3390/ma11010150

**Published:** 2018-01-17

**Authors:** Michihiro Yamada, Yuichi Fujita, Shinya Yamada, Kentarou Sawano, Kohei Hamaya

**Affiliations:** 1 Graduate School of Engineering Science, Osaka University, 1-3 Machikaneyama, Toyonaka 560-8531, Japan; michihiro@ee.es.osaka-u.ac.jp (M.Y.); yuichifujita141@s.ee.es.osaka-u.ac.jp (Y.F.); yamada@ee.es.osaka-u.ac.jp (S.Y.); 2 Center for Spintronics Research Network, Graduate School of Engineering Science, Osaka University, 1-3 Machikaneyama, Toyonaka 560-8531, Japan; 3 Advanced Research Laboratories, Tokyo City University, 8-15-1 Todoroki, Tokyo 158-0082, Japan; sawano@tcu.ac.jp

**Keywords:** semiconductor spintronics, germanium, spin absorption

## Abstract

We study the influence of the junction size in ferromagnet (FM)/semiconductor (SC) contacts on four-terminal nonlocal spin signals in SC-based lateral spin-valve (LSV) structures. When we use FM/Ge Schottky-tunnel junctions with relatively low resistance-area products, the magnitude of the nonlocal spin signal depends clearly on the junction size, indicating the presence of the spin absorption effect at the spin-injector contact. The temperature-dependent spin signal can also be affected by the spin absorption effect. For SC spintronic applications with a low parasitic resistance, we should consider the influence of the spin absorption on the spin-transport signals in SC-based device structures.

## 1. Introduction

Spin-based electronics (spintronics) are expected for one of the beyond CMOS technologies because of the advantages of nonvolatility, reconstructibility, and low power consumption [1,2,3,4,5]. In the field of semiconductor-based spintronics, electrical spin injection and detection in GaAs have so far been demonstrated [6,7,8,9]. Taking the compatibility with existing Si-based electronic devices into consideration, it is important to electrically inject and detect spins in group-IV semiconductors such as Si [10,11,12,13,14,15,16,17,18] and Ge [19,20,21,22,23,24,25,26,27]. However, due to the large conductivity (spin resistance) mismatch between ferromagnet (FM) and semiconductor (SC) [28], it has been generally recognized that tunnel barriers should be inserted at the FM/SC interface for electrical spin injection and detection in SC [29,30,31]. Actually, many studies have utilized insulating tunnel barriers such as MgO for injecting spins into Si [10,11,12,16,18] and Ge [19,20,21,23].

On the other hand, for the spin injection into nonmagnetic metals (NMs), the size of FM/NM junctions was frequently important because of the spin absorption effect at the FM/NM interface [32,33,34]. In general, the spin resistance of FM is defined as 2λρ/[*S*(1 −$\alpha^{2}$)] [30,32], where *S* is the effective cross-sectional area, α is the spin polarization, and λ and ρ are the spin diffusion length and resistivity, respectively. Owing to the unique values of λ, α, and ρ in FM, reducing the size of *S* enables the increase in the spin resistance of FM, resulting in the suppression of the back flows of spins from NM towards FM [32,33,34]. Yang et al. demonstrated the generation of a giant pure spin current, leading to the magnetization switching of the nanomagnet by reducing the FM/NM junction size [33].

Even in CoFe/MgO/*n*-Si (∼10${}^{19}$ cm${}^{-3}$) systems, Ishikawa et al. indicated the significance of the spin absorption effect [35]. Since relatively large junction (∼200 μm${}^{2}$) sizes were used compared to the case of metallic FM/NM junctions, they took into account the value of the resistance-area product (RA) of the contacts in ranging from 10 to 20 kΩ·μm${}^{2}$ [35]. On the other hand, Dushenko et al. recently reported the spin injection from Py into *n*-Ge (∼10${}^{19}$ cm${}^{-3}$) by using a spin-pumping method through the Ohmic interface with very large contacts (∼10${}^{5}$ μm${}^{2}$) [27]. This means that the spin absorption effect due to the large difference in the spin resistance between Py and Ge is ignored despite the Ohmic junction with a large contact area. From these experimental points of view, the presence of the spin absorption effect at FM/SC interfaces remains an open question.

Until now, by using reliable four-terminal nonlocal voltage measurements [36,37,38], we have demonstrated electrical spin injection/detection in *n*-Ge (∼10${}^{19}$ cm${}^{-3}$) via epitaxial FM/Ge Schottky-tunnel junctions [25,26,39,40], where the values of RA (<0.5 kΩ·μm${}^{2}$) were much smaller than those in FM/MgO/Ge junctions [19,20,21,23]. In this paper, to explore the spin absorption effect at the epitaxial FM/Ge Schottky-tunnel interfaces, we study the junction-size dependence of four-terminal nonlocal spin signals in lateral spin valve (LSV) devices with RA ∼ 0.4 kΩ·μm${}^{2}$. At low temperatures, the magnitude of spin signals evidently depends on the size of the epitaxial FM/Ge Schottky tunnel junctions. Note that the smaller junctions enable us to observe the spin signals at higher temperatures. For SC spintronic applications [41] with a low parasitic resistance, we should consider the influence of the spin absorption on the spin-transport signals in SC-based device structures.

## 2. Samples and Fabrication Procedures

**Figure 1:**
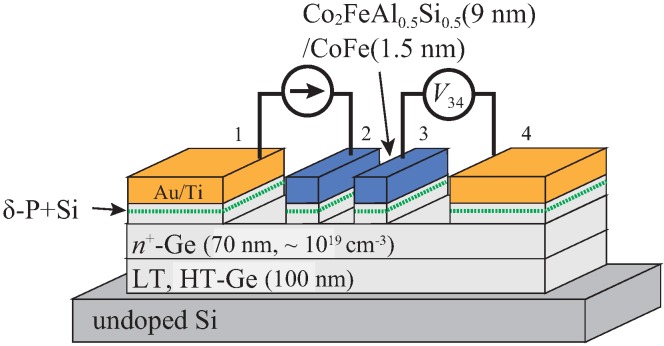
Schematic illustration of the fabricated lateral spin valve with ferromagnet (FM)/Ge Schottky-tunnel contacts. HT: high-temperature-grown; LT: low-temperature-grown.

**Figure 2:**
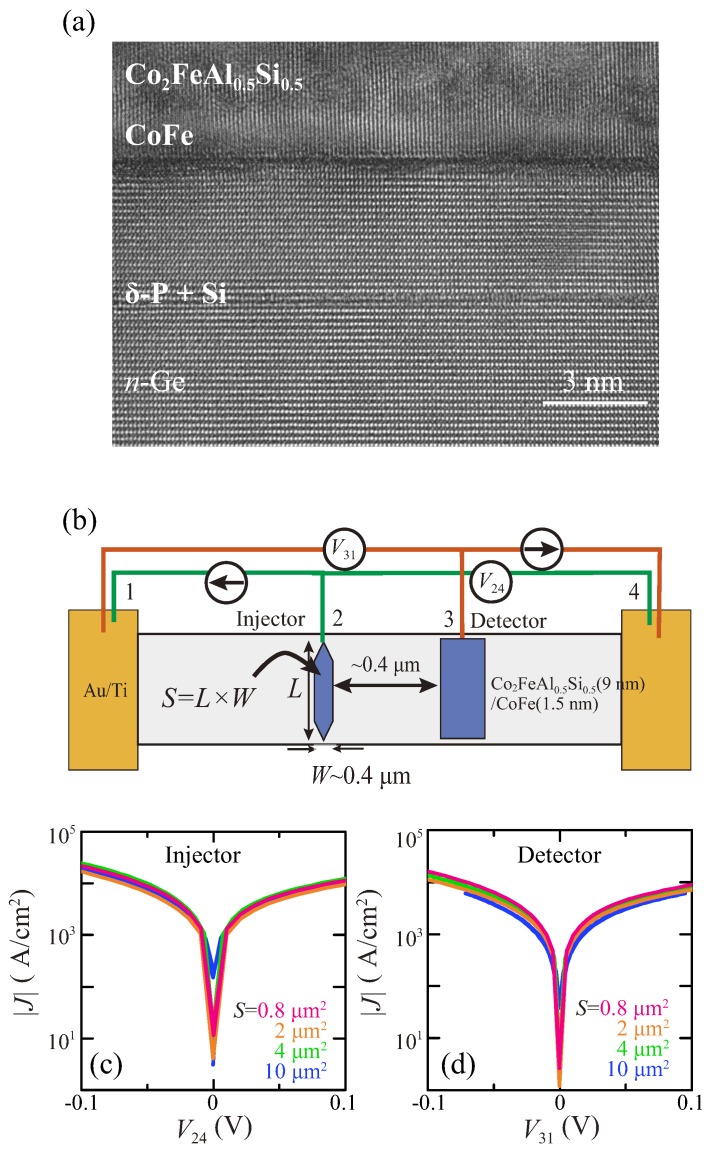
(**a**) Cross-sectional TEM image of the FM/*n*-Ge interface of a measured lateral spin valve (LSV) device; (**b**) Schematic illustration of the terminal configuration for |*J*|–*V* measurements; |*J*|–*V* curves for (**c**) spin injector and (**d**) spin detector with various contact sizes at 30 K.

Ge-based LSV structures with FM/Ge Schottky-tunnel contacts were fabricated as shown in Figure 1. By molecular beam epitaxy (MBE), we first grew an undoped Ge(111) layer (∼28 nm) at 350 ${}^{\circ }$C (LT-Ge) on a commercial undoped Si(111) substrate (ρ ∼ 1000 Ω cm). Next, an undoped Ge(111) layer (∼70 nm) at 700 ${}^{\circ }$C (HT-Ge) was grown on top of the LT-Ge [42]. As the spin transport layer (*n*-Ge), we grew a 70-nm-thick phosphorous (P)-doped *n*-Ge(111) layer (doping concentration ∼10${}^{19}$ cm${}^{-3}$) by MBE at 350 ${}^{\circ }$C on top of the HT-Ge layer. To achieve Schottky tunnel junction, the P δ-doping with an ultra-thin Si insertion was performed between FM and *n*-Ge channel [43]. The P+Si δ-doped Ge layer enables us to promote the tunnel conduction and to precisely adjust the RA values at FM/Ge junctions [44]. Here we used a Co${}_{2}$FeAl${}_{0.5}$Si${}_{0.5}$ (9 nm)/CoFe (1.5 nm) bilayer structure as the FM spin injector/detector, as previously shown in Ref. [39]. If we used highly spin-polarized FM/Ge contacts as a spin injector and detector, we may not have been able to explore the spin absorption effect because of the achievement of the high spin resistance of the FM/Ge contacts. Since Co${}_{2}$FeAl${}_{0.5}$Si${}_{0.5}$ was well known to be a half-metallic material [45], we intentionally inserted a conventional FM, CoFe, between Co${}_{2}$FeAl${}_{0.5}$Si${}_{0.5}$ and Ge. Thus, the large enhancement in the spin resistance of the FM/Ge contacts can be ignored. The growth of the epitaxial FM layers on Ge was already shown elsewhere [25,46,47,48]. Finally, by using electron beam lithography and Ar ion milling, the grown FM/*n*-Ge layers were patterned into the FM contacts with various sizes (*S* = L×W, shown in Figure 2b), ranging from 0.8 μm${}^{2}$ to 10 μm${}^{2}$. The FM contacts had shape-induced anisotropy along the longitudinal direction and almost single magnetic domain in these small sizes. Contact 2 had rhomboid shape to induce the large shape anisotropy, leading to the difference in the magnetization switching field between contact 2 and contact 3. For spin transport measurements, since the external magnetic fields were applied along the longitudinal direction of the contacts, single-domain-like magnetization reversal processes can be expected. Here the P δ-doped layer was removed in the region of the spin-transport channel by using Ar ion milling, as shown in Figure 1.

The edge-to-edge distance between the FM/Ge Schottky-tunnel contacts was designed to be ∼0.4 μm. To fabricate Ohmic contacts as the reference electrodes, we removed the grown Co${}_{2}$FeAl${}_{0.5}$Si${}_{0.5}$/CoFe bilayer by using Ar ion milling techniques. After the removal, we formed the Au/Ti bilayer by electron beam evaporation.

## 3. Results and Discussion

Figure 2a is a cross-sectional transmission electron microscope (TEM) image of a Co${}_{2}$FeAl${}_{0.5}$Si${}_{0.5}$/CoFe/Ge contact in the fabricated LSVs used for spin transport measurements. The abrupt interface between FM and Ge is clearly observed, as well as a P δ-doped layer with an ultra-thin Si insertion. Additionally, we can actually see the inserted CoFe layer between Co${}_{2}$FeAl${}_{0.5}$Si${}_{0.5}$ and Ge.

We first measure current density (|*J*|)–voltage (*V*) characteristics of the high-quality FM/Ge contacts by using three-terminal voltage measurements, which reveal the FM/Ge junction characteristics without the channel, presented in Figure 2b. As mentioned before, the definition of the size of contacts, *S* (=L×W), was also depicted in Figure 2b. Figure 2c,d show |*J*|–*V* curves of the used spin-injector and spin-detector contacts with various *S* at 30 K, respectively.

As previously shown in Ref. [39], we can also see no rectification in the |*J*|–*V* curves, indicating the demonstration of the tunneling conduction of electrons through the FM/Ge interfaces. It should be noted that |*J*|–*V* characteristics for both spin injector and detector contacts are almost the same. This means that the electrical properties of the FM/Ge Schottky-tunnel contacts are uniform irrespective of *S*. From these results, we can precisely adjust the value of RA to be ∼0.4 kΩ·μm${}^{2}$ for all the LSVs used here.

**Figure 3:**
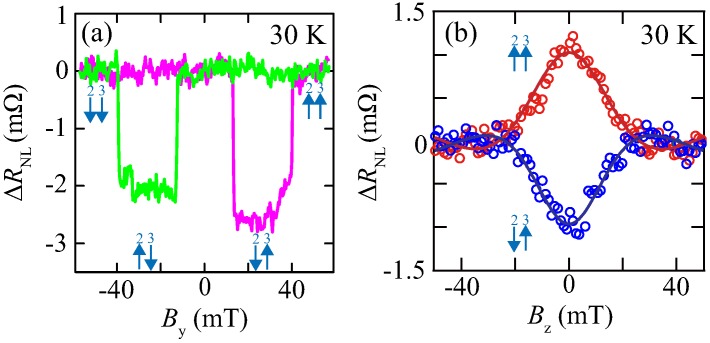
Four-terminal nonlocal (**a**) magnetoresistance curve and (**b**) Hanle-effect curves for the parallel and antiparallel magnetization configurations at 30 K. The solid curves in (**b**) indicate the results fitted to Equation (2) in Ref. [37].

By applying in-plane magnetic fields ($B_{y}$) or out-of-plane magnetic fields ($B_{z}$) to the FM/Ge contacts in an LSV, representative four-terminal nonlocal magnetoresistance ($\Delta R_{\mathrm{NL}}=\Delta V_{34}$/$I_{21}$, in Figure 1) data and nonlocal Hanle-effect curves were recorded at *I* = −1 mA at 30 K, as shown in Figure 3a,b, respectively. We can clearly see hysteretic behavior of the $\Delta R_{\mathrm{NL}}$ depending on the parallel or anti-parallel magnetization configuration between the spin injector and the spin detector in Figure 3a. In Figure 3b, spin precessional behavior in both parallel and antiparallel magnetic configurations can also be observed. From these data, we can recognize that the generation, manipulation, and detection of pure spin currents through *n*-Ge are reliably demonstrated by all electrical means. By using these reliable devices and techniques, we can explore the influence of the spin absorption effect at the FM/Ge interface.

**Figure 4:**
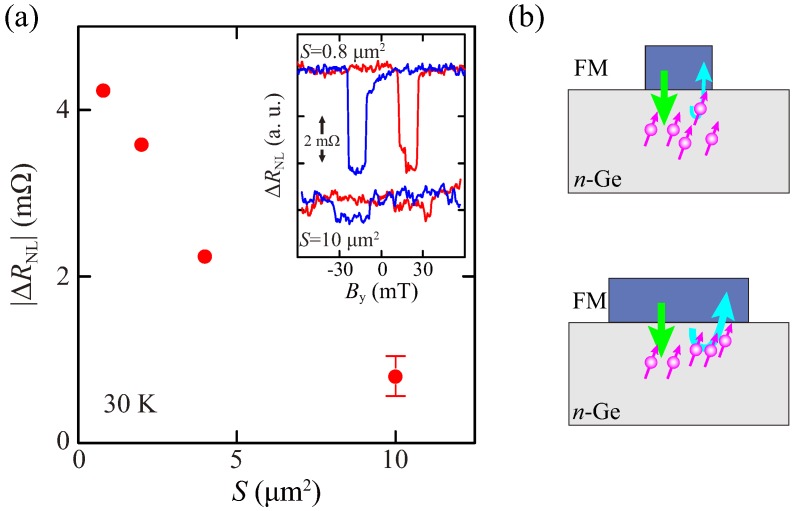
(**a**) *S* dependence of $|\Delta R_{\mathrm{NL}}|$ at 30 K. The inset shows the nonlocal spin signal for the LSVs with *S* = 0.8 and 10 μm${}^{2}$; (**b**) Schematic diagram of spin absorption effect under the small and large FM contacts.

For various LSVs with different *S*, we measured $\Delta R_{\mathrm{NL}}-B_{y}$ curves at 30 K. Figure 4a shows the plot of the magnitude of $\Delta R_{\mathrm{NL}}$, $|\Delta R_{\mathrm{NL}}|$, versus *S*. The inset shows the observed nonlocal spin-valve signals. With increasing *S*, $|\Delta R_{\mathrm{NL}}|$ markedly decreases, although all the LSVs are fabricated from the same epitaxial layers including FM/Ge. Thus, we can neglect the influences of the difference in the spin diffusion length of the *n*-Ge channel and in the quality of the FM/Ge contacts. As shown in the inset, the shape of the $\Delta R_{\mathrm{NL}}-B_{y}$ curve becomes unclear for the LSV with the largest *S*. This means that the influence of *S* on the spin transport in *n*-Ge is more significant than other factors. As mentioned in Section 1, when the spin injection from FM into NM is considered, the spin resistance at the FM contact can be regarded as 2λρ/[*S*(1 −$\alpha^{2}$)] [30,32]. If *S* is changed from 10 μm${}^{2}$ to 0.8 μm${}^{2}$, the spin resistance of the FM contact can be varied by more than by a factor of 10. For our electrical spin injection techniques through FM/Ge Schottky-tunnel contacts, the difference between the RA value of FM/Ge Schottky-tunnel contacts and the spin resistance of Ge is approximately one order of magnitude. Although this condition is satisfied with a condition of the spin injection from FM into SC via tunnel barriers [31,49], we should consider the influence of the spin absorption at the FM/SC interface via tunnel barriers, as previously discussed [35].

**Figure 5:**
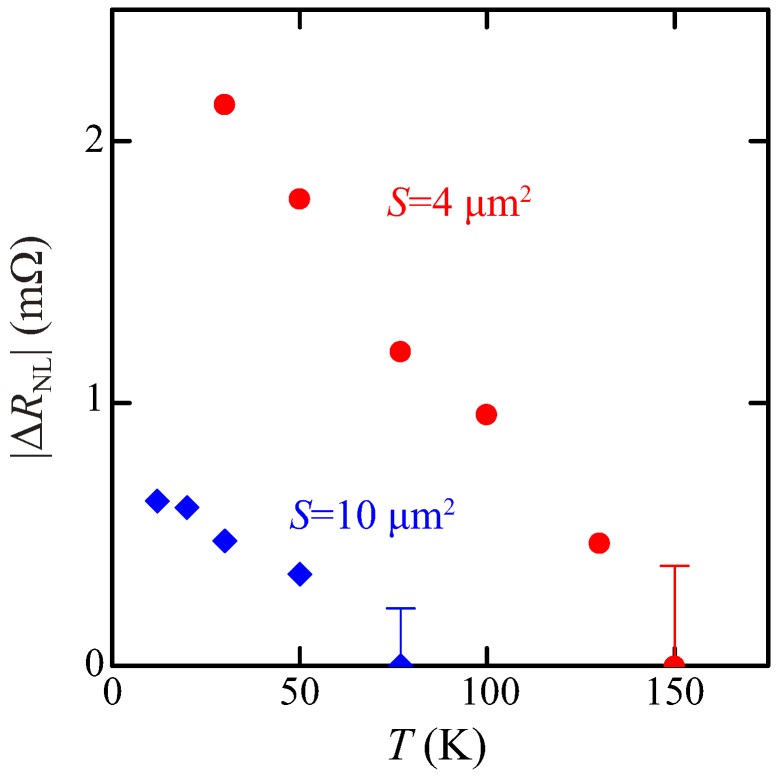
Temperature dependence of spin signal for LSVs with *S* = 4 (circle) and 10 (diamond) μm${}^{2}$.

Figure 5 shows the temperature dependence of $|\Delta R_{\mathrm{NL}}|$ for LSVs with *S* = 4 and 10 μm${}^{2}$. The influence of *S* becomes remarkable when observing the spin signal at higher temperatures. Despite the same spin relaxation mechanism in the used *n*-Ge channel, the spin signals for *S* = 10 μm${}^{2}$ disappear below 100 K, while we can see those for *S* = 4 μm${}^{2}$ above 100 K. The same behavior was observed in the spin accumulation in *n*-Si detected by the three terminal method [35]. The features in Figure 5 can also be interpreted by the difference in the spin absorption at the spin injector in the FM/Ge Schottky-tunnel contacts.

Finally, we comment on the importance of geometrical factors of FM contacts in SC spintronic applications. In general, the low RA contacts are first required to reduce the parasitic resistance, leading to the low power consumption. In addition, according to a conventional spin drift-diffusion model [31,49], the ratio of RA (=r${}_{b}$) to r${}_{\mathrm{SC}}$ (=ρ${}_{\mathrm{SC}}$ × λ${}_{\mathrm{SC}}$) is important to obtain a large magnetoresistance (MR) ratio, where ρ${}_{\mathrm{SC}}$ and λ${}_{\mathrm{SC}}$ are the resistivity and spin diffusion length of the semiconductor channel, respectively. In this study, because r${}_{\mathrm{SC}}$ can be estimated to be ∼0.01 kΩ·μm${}^{2}$ at 30 K, RA/r${}_{\mathrm{SC}}$ is ∼40. For reaching an optimal condition (RA/r${}_{\mathrm{SC}}∼$ 1), we should further reduce the value of RA. If the value of RA was reduced down to 0.01 kΩ·μm${}^{2}$, the spin absorption could be made even more significant, as mentioned above. Thus, the decrease in *S* is more effective for SC spintronic applications to achieve highly efficient spin injection from FM into SC without increasing the value of RA.

## 4. Conclusions

We studied the influence of the junction size in ferromagnet (FM)/semiconductor (SC) contacts on four-terminal nonlocal spin signals in SC-based lateral spin-valve structures. When we used FM/Ge Schottky-tunnel junctions with relatively low RA, the magnitude of the nonlocal spin signal depended clearly on the junction size, meaning the presence of the spin absorption effect at the spin-injector contact. The temperature-dependent spin signal can also be affected by the spin absorption effect. For SC spintronic applications with a low parasitic resistance, we should consider the influence of the spin absorption on the spin-transport signals in SC-based device structures.
